# Molecular-based classification of endometrial carcinoma in Northern Thailand: impact on prognosis and potential for implementation in resource-limited settings

**DOI:** 10.1186/s12905-023-02677-6

**Published:** 2023-11-14

**Authors:** Wiyada Dankai, Tip Pongsuvareeyakul, Phichayut Phinyo, Chontichaporn Tejamai, Chinachote Teerapakpinyo, Chalong Cheewakriangkrai, Suree Lekawanvijit, Sumalee Siriaunkgul, Surapan Khunamornpong

**Affiliations:** 1 Department of Pathology, Faculty of Medicine, Chiang Mai University, 110 Inthawaroros Road, Sri Phum District, Muang Chiang Mai, Chiang Mai, Chiang Mai, 50200 Thailand; 2 Gynecologic Cancer Research Center, Faculty of Medicine, Chiang Mai University, Chiang Mai, 50200 Thailand; 3 Center for Clinical Epidemiology and Clinical Statistics, Faculty of Medicine, Chiang Mai University, Chiang Mai, 50200 Thailand; 4 Chulalongkorn GenePRO Center, Research Affairs, Faculty of Medicine, Chulalongkorn University, Bangkok, 10330 Thailand; 5 Department of Obstetrics and Gynecology, Faculty of Medicine, Chiang Mai University, Chiang Mai, 50200 Thailand

**Keywords:** Endometrial carcinoma, Molecular classification, *POLE* mutation, Resource-limited setting, Management

## Abstract

**Background:**

Endometrial carcinoma is molecularly categorized into four subgroups: *polymerase-E* exonuclease domain-mutant (POLE-mut), mismatch repair-deficient (MMR-d), p53-abnormal (p53-abn), and no specific molecular profile (NSMP). This classification scheme has been included into clinical recommendation for post-operative risk-based management, although there have been few Asian studies on this topic. The present study aimed to evaluate the prevalence and clinical outcomes of endometrial carcinoma using this classification in Northern Thailand and the feasibility of implementation in resource-limited settings.

**Methods:**

Endometrial carcinomas from hysterectomy specimens were classified using immunohistochemistry for MMR proteins and p53, as well as *POLE* mutation testing. Clinicopathological variables and outcomes were analyzed. The costs of the molecular information-based approach were compared to those incurred by the conventional approach (without molecular classification).

**Results:**

Of 138 patients, 52.9% in the NSMP subgroup, 28.2% were in the MMR-d, 13.8% in the p53-abn, and 5.1% in the POLE-mut. After adjusting for other variables, patients with POLE-mut showed the most favorable outcomes, while those with p53-abn had the poorest survival. When estimating the costs for post-operative management, the use of molecular classification resulted in a 10% increase over the conventional approach. However, the cost increased only by 1% if only *POLE* testing was used to identify patients for treatment omission.

**Conclusion:**

In Northern Thailand, endometrial carcinoma had comparable subgroup distribution and prognostic implications to previous reports, supporting the implementation of management guidelines that incorporate molecular information. In resource-limited settings, at least *POLE* mutation testing in early-stage patients should be considered.

## Background

Endometrial carcinoma is the sixth most common cancer in females worldwide and the third most common cancer in the female genital tract in Southeast Asia, with an annual incidence of 32,000 new cases [2]. The major factors influencing postsurgical treatment decision are surgical staging and pathological findings [3]. Histological type has traditionally been an important parameter for risk stratification [3], but it has been shown to be poorly reproducible, with a significant disagreement rate as high as 35%, particularly in high-grade carcinoma [4, 5].

The Cancer Genome Atlas Research Network has identified four molecularly distinct subgroups of endometrial carcinoma with different clinical outcomes: *POLE* ultramutated (*polymerase-E* exonuclease domain-mutant: POLE-mut), microsatellite instability hypermutated (mismatch repair-deficient: MMR-d), copy number high (p53-mutant/-abnormal: p53-abn), and copy number low (no specific molecular profile: NSMP) [6]. This new approach provided better diagnostic reproducibility than the traditional histologic typing and grading, thus improving the precision of prognostication and therapeutic decision-making [4, 5].

The European Society for Medical Oncology (ESMO), European Society for Radiotherapy & Oncology (ESTRO), and European Society of Gynaecological Oncology (ESGO) consensus guidelines for risk-based management of endometrial carcinoma have duly incorporated this molecular-based classification and introduced significant differences in management between each molecular subgroups [9]. This paradigm shift has also been reflected in the updated 2023 International Federation of Gynecology and Obstetrics (FIGO) staging system [10]. Besides, the extremely high mutation burdens seen in POLE-mut and MMR-d tumors, which greatly increase antigenicity, make immunotherapy an especially appealing treatment option [11]. While some may be concerned about the economic implications of these molecular studies, particularly in resource-constrained settings, studies have shown that TCGA-based molecular categorization can be seamlessly integrated into routine clinical practice by using immunohistochemistry (IHC) for MMR proteins and p53, and *POLE* mutation testing, rather than comprehensive molecular testing [7, 8].

The present study aimed to assess the prevalence and clinical outcomes among the different molecular subgroups of endometrial carcinoma in Northern Thailand. We also compared the financial costs of the molecular information-based strategy to those of the conventional approach.

## Materials and methods

### Study population

The study cohort included women diagnosed with endometrial carcinoma at Chiang Mai University Hospital during January 2015 to December 2017. Inclusion criteria were patients with informed consent, available hysterectomy specimens for evaluation, and available clinical information and pathological results. We excluded individual without histologic materials and formalin-fixed paraffin-embedded (FFPE) tissue blocks and those with suspected cervical cancer. In cases where there was suspicion of cervical cancer extending to the endometrium, IHC panel was performed, including p16, estrogen receptor (ER), progesterone receptor (PR), and/or vimentin.

Pathology reports of eligible cases were reviewed (WD and TP). Pathologic findings included tumor size, histological type, extent of uterine wall invasion, presence of lymphovascular space invasion, endocervical stromal invasion, and lymph node metastasis. To confirm the tumor origin and the histological classification, a gynecologic pathologist (TP) reviewed the histological slides in accordance with the current World Health Organization Classification (WHO) guidelines [12]. In cases where there were diagnostic discrepancies, a consensus was achieved through consultation with another gynecologic pathologist (SK). In each case, 1–2 representative histologic slides with the highest-quality tumor preservation were selected, and the corresponding FFPE blocks were retrieved for further testing.

Clinical information, comprising patient age, FIGO stage, and follow-up data up to January 2023, was obtained from electronic medical records and Chiang Mai Cancer Registry. Disease progression was defined as tumor recurrence or progression confirmed by imaging studies or histology. Progression-free survival (PFS) denoted the duration from the date of surgery to the date of disease progression, while overall survival (OS) was defined as the interval between the date of surgery and the date of last follow-up or death from any cause.

### Immunohistochemistry for mismatch repair proteins and p53

Immunohistochemical staining was performed using BenchMark ULTRA IHC/ISH platform (Ventana Medical Systems, Roche Diagnostics, Tucson, AZ, USA), following the manufacturers’ instructions. To assess MMR protein status, a streamlined approach using two key IHC markers (PMS2 and MSH6) were employed instead of the full panel of four markers (MLH1, PMS2, MSH2, and MSH6). This approach was chosen based on established evidence demonstrating its cost-effectiveness and reliability [13, 14]. Primary monoclonal antibodies against PMS2 (A16-4 Ventana clone ready to use; Optiview kit revelation with amplification) and MSH6 (SP93 Ventana clone ready to use; Optiview kit revelation) were used. Evaluation of MMR protein status was performed in cases exhibiting nuclear positivity in internal non-neoplastic tissues, serving as an internal positive control. The loss of MMR protein expression was defined as the complete absence of nuclear staining in tumor cells, while internal non-neoplastic tissues retained their staining. Cases showing the loss of at least one MMR protein expression were classified as MMR-d.

For p53 expression, we used a primary monoclonal antibody against the p53 protein (DO-7 clone, DAKO, dilution 1:100). The results were categorized into two groups: wild-type expression and abnormal expression. Specifically, wild-type p53 expression was characterized by a mixture of tumor cells displaying variable nuclear staining. Abnormal p53 expression included three main patterns: the diffuse pattern (strong positivity in at least 80% of tumor nuclei), the null pattern (absence of tumor nuclear staining), and the cytoplasmic pattern (unequivocal cytoplasmic staining of tumor cells accompanied by variable nuclear staining) [15]. Cases with any type of abnormal p53 expression were classified as p53-abn.

### DNA extraction and *POLE* mutation analysis

Genomic DNA was extracted from three ribbons of five µm-thick FFPE sections selected from regions with ≥ 60% tumor nuclei and < 10% necrosis, the extraction was performed according to the manufacturer’s recommendations using the QIAamp DNA FFPE Tissue kit (Qiagen, Toronto, ON, Canada). The concentration and purity of the DNA were quantified using a UV spectrophotometer (Nanodrop 2000, Thermo Fisher Scientific, Waltham, MA, USA) with A_260/280_ and A_260/230_ ratios that were expected to be within the range of 1.80–2.30. *GAPDH* was used as a reference gene for quantitative DNA analysis, and cases with no *GAPDH* amplification were not further processed for *POLE* mutation sequencing.

The *POLE* gene exons 9–14 were then amplified using previously published primers [16]. Following confirmation of the presence of target amplicons and absence of non-specific amplification products, bi-directional Sanger sequencing was performed according to standard protocols using BigDye™ Terminator v 3.1 Cycle Sequencing Kit (Thermo Fisher Scientific Baltics UAB, Vilnius) and Seqstudio genetic analyzer (Lifetechnologies holdings Pte Ltd, Singapore). Using the NM_006231.4 reference sequence, all sequences were evaluated for the presence of pathogenic mutations, including five hotspot mutations (P286R, S297F, V411L, A456P, and S459F) and other rare mutation types [12]. To confirm the results, new PCR products from all mutation-positive samples were re-sequenced, and samples containing confirmed pathogenic mutations were classified as POLE-mut.

### Molecular-based classification of endometrial carcinoma

The tumors in our cohort then were categorized using the Proactive Molecular Risk Classifier for Endometrial Cancer (ProMisE) algorithm, which has been proposed to be appropriate for clinical implementation [17]. This model follows a stepwise approach, using MMR IHC, *POLE* sequencing, and p53 IHC. Cases with suboptimal DNA quality were excluded.

### Cost estimation for the application of molecular-based classification

We acquired cost information for IHC and adjuvant therapy from our institution’s cost announcements. The cost of *POLE* mutation analysis (Sanger sequencing for five hotspot mutations) was calculated using the costs of prior in-house testing at our institution. First, for each patient, we evaluated the direct medical costs for adjuvant therapy according to ESMO/ESTRO/ESGO recommendations [9]. These individual costs were summed to provide a reference cost. We then estimated the costs for the molecular-based strategy by combining the cost of IHC and/or molecular testing with the cost of adjuvant treatment. Finally, we compared the reference cost to the estimated cost derived after incorporating molecular information and management modification. Only patients with stage I-II were included in this comparison because molecular findings caused substantial management changes in this group, such as the omission of adjuvant therapy in the POLE-mut subgroup or the addition of chemo-radiation therapy in the p53-abn subgroup [9]. We also assessed the costs of different strategies for EC molecular-based approach, including WHO algorithm [12], ProMisE model [17], and *POLE* mutation testing alone.

### Statistical analysis

All statistical analyses were performed using STATA version 16 (STATA Corp., Texas, USA). Continuous data were compared using one-way analysis of variance (ANOVA), while categorical data were assessed using the Chi-square test to analyze univariable associations of molecular subtypes of endometrial carcinoma. Kaplan-Meier methods and log rank test were used to explore the association between baseline prognostic variables and survival endpoints. A p value < 0.05 was considered statistically significant.

We conducted a flexible parametric regression analysis to elucidate the association of molecular subgroups and patient’s OS and PFS [18]. For patients who had not experienced an event, they were censored at their last follow-up. To account for confounding factors, we generated a confounder summary score (CFS). This score was constructed by incorporating baseline prognostic variables (i.e., age, FIGO stage, histological type, degree of uterine wall invasion, and lymphovascular space invasion) into the flexible parametric model, excluding the molecular results [19]. Subsequently, we estimated the model’s linear predictors to generate the CFS, which was then included in the main flexible parametric model to compute adjusted restricted mean survival time (RMST) for each molecular subgroup at five-year follow-up interval [20].

## Results

### Clinicopathological and molecular features

**Fig. 1:**
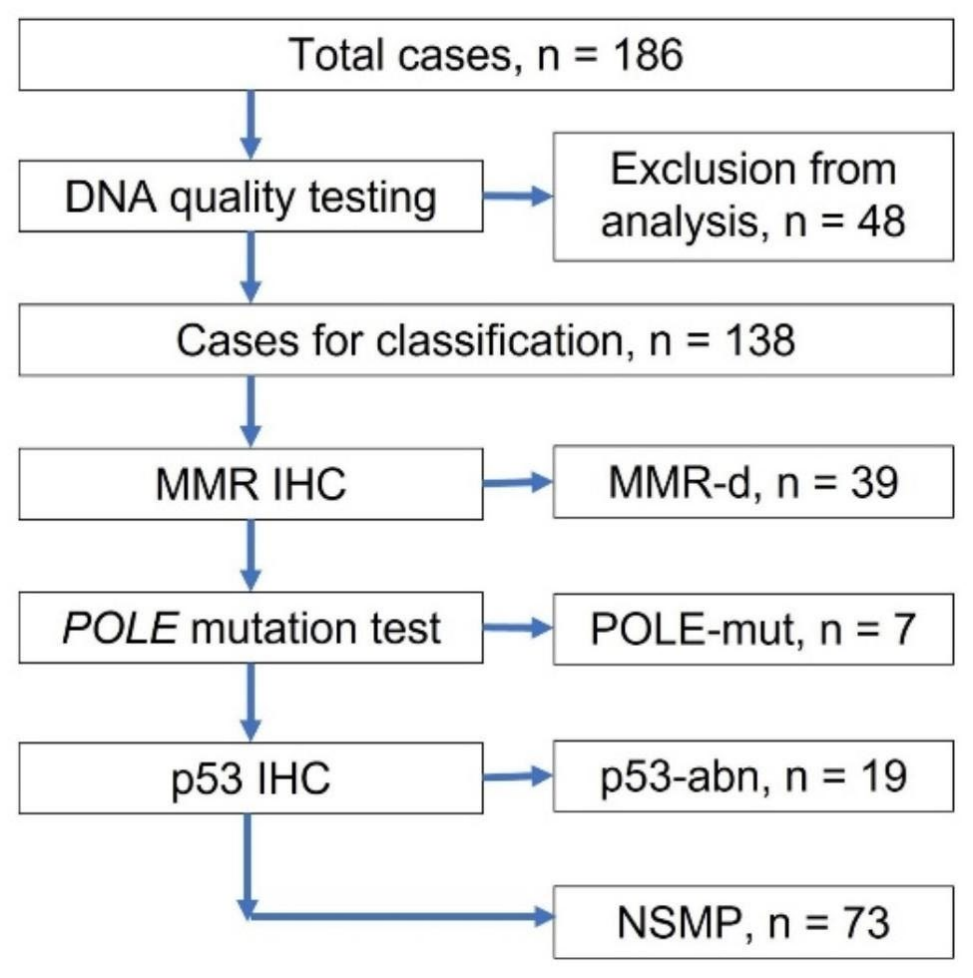
Consort flow diagram for molecular-based classification of endometrial carcinoma

Out of the 186 cases initially recruited, 48 (25.8%) were excluded from the analysis due to poor DNA quality (Fig. 1). The remaining 138 patients had a mean age of 57.2 years (range 25–81). The majority of patients (52.9%) were in the NSMP subgroup, followed by MMR-d (28.2%), p53-abn (13.8%), and POLE-mut (5.1%).

There were significant differences in histological types (p < 0.001), degree of uterine wall invasion (p = 0.007), and FIGO stage (p = 0.011) among molecular subgroups (Table 1). The p53-abn subgroup showed predominantly non-endometrioid histology when compared to the POLE-mut subgroup (p = 0.017), the MMR-d subgroup (p < 0.001), and the NSMP subgroup (p < 0.001). The p53-abn subgroup had a higher incidence of uterine serosal involvement than the POLE-mut (p = 0.013), the MMR-d (p = 0.048), and the NSMP subgroup (p < 0.001). Serous adenocarcinoma was the most common histologic type among non-endometrioid cases in the p53-abn subgroup (10 of 12; 83.4%).

**Table 1:** Comparison of clinical and pathological features of molecular subgroups of 138 endometrial carcinoma patients

	No. of Patients					
	Total	MMR-d	POLE-mut	p53-abn	NSMP	P value
No. of Patients	138 (100%)	39 (28.2%)	7 (5.1%)	19 (13.8%)	73 (52.9%)	
**Age at time of diagnosis**: mean (range)	57.15 (25–81)	58.46 (37–78)	57.29 (42–66)	60.79 (29–81)	55.49 (25–75)	0.584
**FIGO Stage**						**0.011**
I –II	80 (58.0%)	25 (64.1%)	5 (71.4%)	5 (26.3%)	45 (67.6%)	
III-IV	58 (42.0%)	14 (35.9%)	2 (28.6%)	14 (73.7%)	28 (32.4%)	
**Histological type**						**< 0.001**
Endometrioid grade 1–2 (low-grade)	81 (58.7%)	25 (64.1%)	4 (57.1%)	0 (0.00)	52 (71.2%)	
Endometrioid grade 3 (high-grade)	39 (28.3%)	12 (30.8%)	3 (42.9%)	7 (36.8%)	17 (23.3%)	
Non-endometrioid	18 (13.0%)	2 (5.1%)	0 (0.0%)	12 (63.2%)	4 (5.5%)	
**Mean tumor size in cm** **(± SD)**	4.9 (± 3.1)	5.1 (± 3.4)	4.5 (± 2.0)	4.9 (± 2.1)	4.8 (± 3.2)	0.967
**Degree of uterine wall invasion**						**0.007**
< 50%	72 (52.2%)	20 (51.3%)	3 (42.9%)	9 (47.4%)	40 (54.8%)	
> 50%	46 (33.3%)	13 (33.3%)	4 (57.1%)	2 (10.5%)	27 (37.0%)	
Invasion through serosa	20 (14.5%)	6 (15.4%)	0 (0.0%)	8 (42.1%)	6 (8.2%)	
**Lymphovascular space invasion**						0.669
No	60 (43.5%)	15 (38.5%)	4 (57.1%)	7 (36.8%)	34 (46.6%)	
Yes	78 (56.5%)	24 (61.5%)	3 (42.9%)	12 (63.2%)	39 (53.4%)	
**Endocervical stromal invasion**						0.960
No	119 (86.2%)	33 (84.6%)	6 (85.7%)	17 (89.5%)	63 (86.3%)	
Yes	19 (13.8%)	6 (15.4%)	1 (14.3%)	2 (10.5%)	10 (13.7%)	
**Lymph node metastasis**						0.195
No	77 (72.0%)	24 (77.4%)	5 (83.3%)	5 (45.5%)	43 (72.9%)	
Yes	30 (28.0%)	7 (22.6%)	1 (16.7%)	6 (54.5%)	16 (27.1%)	
Lymph node not removed	31	8	1	8	14	

### Validation of the prognostic value of molecular-based classification

**Fig. 2:**
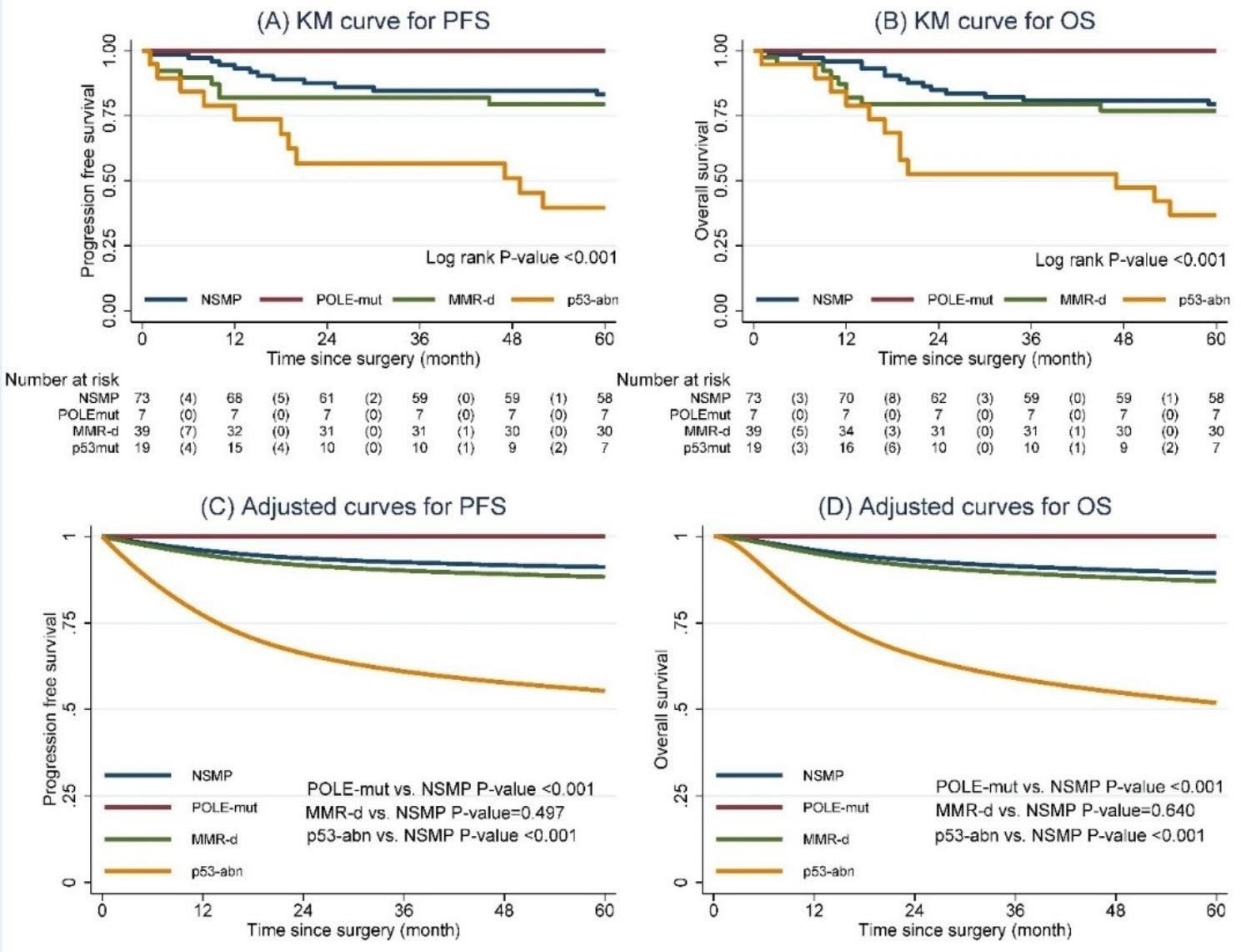
Survival curves for molecular subgroups of endometrial carcinoma. (**A**) Kaplan-Meier (KM) curves for progression free survival (PFS). (**B**) KM curves for overall survival (OS). (**C**) Adjusted survival curve from flexible parametric survival model for PFS. (**D**) Adjusted survival curve from flexible parametric survival model for OS.

All 138 patients were followed for a median of 70 months (range 1–95, interquartile range 69–85). All patients who survived without disease progression were followed for at least 60 months. The Kaplan-Meier analysis and Log-rank test revealed significant differences in PFS and OS between the four molecular subgroups (p < 0.001 for both) (Fig. 2A and B). The flexible parametric survival model’s adjusted survival curve yielded similar results (Fig. 2C and D). There was no disease progression or death in any of the seven patients in the POLE-mut subgroup (duration 64–87 months). Table 2 compares the RMST for PFS and OS at 5 years across the four categories. Compared to the NSMP subgroup, the POLE-mut subgroup had longer PFS and OS (p < 0.001 for both), while the p53-abn subgroup had shorter PFS and OS (p < 0.001 for both).

**Table 2:** Adjusted restricted mean overall and progression free survival time (RMST) of four molecular subgroups of endometrial carcinoma at 5 years

	Progression free survival				Overall survival			
Molecular subgroup	No. of patients at risk	Adjusted RMST^a^ (95% CI) (months)	RMST difference (95% CI) (months)	p value	No. of patients at risk	Adjusted RMST^a^ (95% CI) (months)	RMST difference (95% CI) (months)	p value
NSMP (n = 73)	58	53.2 (49.5, 56.8)	Reference		58	52.0 (48.2, 55.7)	Reference	
POLE-mut (n = 7)	7	60.0 (60.0, 60.0)	+ 6.8 (3.2, 10.5)	< 0.001	7	60.0 (60.0, 60.0)	+ 8.0 (4.3, 11.7)	< 0.001
MMR-d (n = 39)	30	51.1 (45.4, 56.8)	-2.1 (-8.0, 3.9)	0.497	30	50.3 (44.7, 56.0)	-1.7 (-8.7, 5.3)	0.640
p53-abn (n = 19)	7	29.5 (16.8, 42.2)	-23.6 (-36.7, -10.5)	< 0.001	7	28.9 (17.4, 40.4)	-23.0 (-35.0, -11.1)	< 0.001

Abbreviations: CI, confidence interval; RMST, restricted mean survival time

^a^ Adjusted for age, FIGO stage, histological type, degree of uterine wall invasion, and lymphovascular space invasion

### Estimated direct costs associated with the application of molecular-based classification

The omission of unnecessary adjuvant therapy in the POLE-mut subgroup resulted in a cost saving, whereas the addition of chemotherapy for the p53-abn subgroup resulted in a cost increase. Table 3 compares the costs of post-operative management in different approaches. The molecular-based approach cost more than the conventional approach (9.9% by WHO algorithm, 7.9% by ProMisE approach). Such increase was mainly due to the expenses related to the tests and additional chemotherapy for p53-abn subgroup, calculated as (cost of tests + chemotherapy) – (omitted cost of adjuvant therapy). In contrast, testing for the *POLE* mutation alone resulted in only a 1.0% increase over the conventional cost, calculated as (costs of tests) – (omitted cost of adjuvant therapy).

**Table 3:** Comparison of costs for adjuvant therapy and testing for molecular-based classification in 80 patients with early-stage endometrial carcinoma (Thai currency: Baht)

	Conventional approach (reference)	Molecular approaches on all 80 patients			Molecular approaches on selected patients^a^		
		WHO algorithm (estimated values)	ProMisE approach	POLE alone	WHO algorithm (estimated values)	ProMisE approach	POLE alone
**No. of patients in risk group**							
Low	30	33	33	33	33	33	33
Intermediate	18	14	14	17	14	14	17
High-intermediate	29	27	27	27	27	27	27
High	3	6	6	3	6	6	3
Testing performed (No. of patients)		POLE (80) MMR (75) p53 (50)	MMR (80) POLE (55) p53 (50)	POLE (80)	POLE (50) MMR (47) p53 (30)	MMR (50) POLE (33) p53 (30)	POLE (50)
Costs for testing (Baht)	0	495,000	389,600	360,000	310,840	236,600	225,000
Changes in therapy (No. of patients)		• Treatment omission (3 POLE-mut) • Chemotherapy added (3 p53-abn)	• Treatment omission (3 POLE-mut) • Chemotherapy added (3 p53-abn)	• Treatment omission (3 POLE-mut)	• Treatment omission (3 POLE-mut) • Chemotherapy added (3 p53-abn)	• Treatment omission (3 POLE-mut) • Chemotherapy added (3 p53-abn)	• Treatment omission (3 POLE-mut)
Changes in therapy costs (Baht)	0	+ 12,288	+ 12,288	-307,400	+ 12,288	+ 12,288	-307,400
Cost for post-operative management (Baht)^b, c^	5,099,366 (reference)	**5,606,654**	**5,501,254**	**5,151,966**	**5,422,494**	**5,348,254**	**5,016,966**
**Percentage of cost change**	0	**+ 9.9%**	**+ 7.9%**	**+ 1.0%**	**+ 6.3%**	**+ 4.9%**	**-1.6%**

^a^ Testing in selected 50 patients who had at least intermediate risk by conventional risk stratification approach

^b^ [Cost for post-operative management] = [Total costs of adjuvant therapy calculated for all 80 patients] – [Total costs of testing by the approach method]

^c^ Treatment costs for each risk group: intermediate = 53,400 Baht (vaginal brachytherapy); high-intermediate = 127,000 Baht (external beam radiotherapy and vaginal brachytherapy); high = 151,722 Baht (chemotherapy and radiation therapy)

Using the conventional approach, 30 of 80 patients were categorized as low-risk (stage IA, low-grade endometrioid histology, and negative or focal lymphovascular space invasion). The integration of molecular data had no effect on these patient’s risk classification or treatment decisions. The molecular data was deemed necessary only for the remaining 50 patients who were classified as at least intermediate risk. When only these 50 patients were considered, the estimated cost of the strategy using POLE testing alone was 1.6% lower than the reference cost.

## Discussion

We found that the distribution of molecular subgroups in endometrial carcinomas was consistent with previous reports (Table 4) [21–27], with the majority falling into the NSMP subgroup (39–64%), followed by the MMR-d (17–39%), the p53-abn (8–21%), and the POLE-mut (4-13.6%). Our findings also support the prognostic significance of molecular-based classification when applied to the Northern Thai population, indicating its broad applicability. Similar to previous reports [26, 28], patients in the POLE-mut subgroup exhibited most favorable survival outcomes, while the p53-abn subgroup had the worst prognosis. These findings support the use of the recent ESMO/ESTRO/ESGO management guidelines in Northern Thailand.

**Table 4:** Comparison of the previously reported distribution of molecular subgroups of endometrial carcinoma

Authors (publication year)	Country/region, No. of patients	MMR-d (%)	POLE-mut (%)	p53-abn (%)	NSMP (%)
Talhouk et al. (2015) [21]	North America (Canada), n = 143	28.7	8.4	17.5	44.1
Stelloo et al. (2016) [22]	Europe, n = 834	26.3	5.9	8.9	59
Cosgrove et al. (2018) [23]	North America (USA), n = 982	38.6	4.0	8.5	48.9
Kommoss et al. (2018) [24]	Europe (Germany), n = 452	28.1	9.3	12.2	50.4
H et al. (2021) [25]	Oceania (New Zealand), n = 88	17.1	9.1	10.2	63.6
Kim et al. (2022) [27]	Asia (Korea), n = 240	19.6	10.8	17.1	52.5
Asami et al. (2023) [26]	Asia (Japan), n = 265	26.4	13.6	21.1	38.9
Present study	Asia (Thailand), n = 138	28.2	5.1	13.8	52.9

All seven POLE-mut tumors in our cohort had endometrioid histotype, and 42.9% of them were FIGO grade 3, which aligns with the findings in a recent systematic review (43.4%) [28]. Without *POLE* mutation testing, patients with grade 3 tumors would typically be classified as having at least an intermediate risk, which warrants adjuvant therapy [9]. Despite their high histologic grade, patients with POLE-mut tumors had a much lower recurrence rate when compared to the other subgroups [26]. The favorable prognosis of POLE-mut tumors provides a compelling rationale for avoiding adjuvant therapy in early-stage patients, as such treatment is unlikely to improve their outcomes [11, 26].

The p53-abn subgroup, on the other hand, is the most aggressive, necessitating adjuvant therapy. Serous histology is seen in a significant proportion of tumors in this subgroup. It should be noted that serous adenocarcinoma has a broad histological spectrum and is sometimes confused with low-grade endometrioid carcinoma [28]. In cases where molecular testing is not performed, p53 IHC may be useful for screening for p53-abn endometrial carcinoma in tumors with high-grade histology or endometrioid carcinoma with high nuclear grade or overlapping features with serous adenocarcinoma [28]. However, p53-abn tumors may account for 2 to 5% of low-grade endometrioid carcinomas [12], and patients with these tumors may benefit from more aggressive treatment [12]. The 2023 FIGO staging system designated stage I-II low-grade endometrioid endometrial carcinoma with p53-abn as stage IICm_p53abn_ [10], and, as a result, p53 IHC screening is likely to be beneficial in all patients with early-stage disease.

MMR-d and p53-abn may be found in 9.8% and 15.7% of POLE-mut tumors, respectively, since *POLE* mutations cause multiple subsequent mutations, including MMR and *p53* genes [29]. Without *POLE* mutation testing, some POLE-mut tumors in our study could have been misclassified as MMR-d. This is significant because *POLE* mutations have been shown to be a prognostic driver even when MMR deficiency or *p53* abnormalities are present [30].

One of the challenges of implementing molecular-based classification is the cost of molecular testing [11], which is especially significant in resource-limited settings. While we anticipated additional costs, our findings suggest that incorporating *POLE* mutation testing into early-stage patient management may not result in a significant increase in overall costs when compared to the conventional approach. This was primarily due to the fact that omitting adjuvant therapy in POLE-mut patients saved money. Interestingly, by carefully selecting patients with at least intermediate risk, doing *POLE* mutation testing alone could result in a 1.6% cost reduction, suggesting that testing for *POLE* mutations could be useful in early-stage patients, even in resource-constrained settings. In such cases, a single molecular testing center could serve as a reference laboratory. As technology advances and the volume of testing increases, the cost tends to decrease.

*POLE* mutation status can be determined using DNA sequencing techniques, such as next-generation sequencing, Sanger sequencing, or hotspot mutation analysis [12]. In our case, the cost of next-generation sequencing testing was estimated to be 3–4 times that of five hotspot Sanger sequencing. Recently, quantitative PCR and droplet digital PCR to detect *POLE* mutations have been developed [27]. Table 5 compares the cost of testing, detected mutations, advantages, and limitations of each method, including Sanger sequencing in exon 9–14 used in this study.

**Table 5:** Comparison of detection techniques for pathogenic *POLE* mutation in terms of costs, detected mutations, advantages, and limitations

Detection technique	Cost of test	Detected mutations	Advantages	Limitations
Next-generation sequencing [32, 33]	Highest	Full range^a^	- High sensitivity of detection - Simultaneous testing for multiple mutation types (high throughput)	- Requirement of high technology equipment and expert bioinformatics in the interpretation - Time-consuming
Quantitative PCR [31]	Intermediate	11 mutations	- High sensitivity of detection - Rapid processing	- Limited data - Further validation needed
Droplet digital PCR assay [27]	Intermediate	Five hot spot mutations	- Rapid processing	- Limited data - Further validation needed
Sanger sequencing in exon 9,10,11,12,13, and 14 ^b^ [34]	Intermediate	Full range^a^	- High accuracy - Rather simple technique	- Limit of detection; not suitable for samples with a low proportion of mutation sequence - Not suitable for testing a large volume of samples
Sanger sequencing for five hotspot mutations	Lowest	Five hot spot mutations, accounting for 95% of POLE-mut cases [31]	- High accuracy - Rather simple technique	- Limit of detection; not suitable for samples with a low proportion of mutation sequence - Not suitable for testing a large volume of samples

^a^ Full mutation range includes 12 recognized pathogenic *POLE* mutations in exonuclease domain.

^b^ Method used in this study.

In this new era of endometrial carcinoma treatment, molecular-based classification is becoming increasingly important in determining the best therapeutic approach. The treatment is rapidly evolving, driven by the development of novel strategies [35–38]. For example, the discovery of new mutational pathways, such as the PI3K-AKT or FBXW7-FGFR2 pathways, could become a potential option for targeted therapy in patients with poor prognosis, particularly in the p53-abn or NSMP subgroups and non-endometrioid histotype [37]. In addition, integrating preoperative radiomic analysis using MRI findings with molecular testing has the potential to improve risk stratification and enable more personalized treatments [38].

This is the first study to look into the molecular data of endometrial carcinoma in Southeast Asia, and it provides information on the prevalence and outcomes of each molecular subgroup in this population. Because molecular-based classification has become a current topic of interest, this study focuses on its application in routine clinical practice in resource-limited settings. Limitations of our study include the study’s retrospective nature, small sample size, study design, and proportion of cases with suboptimal DNA preservation (25.8% of recruited cases), which may all affect the distribution of the subgroups. The DNA preservation factor could be attributed, at least in part, to our tropical environment and/or the duration of FFPE block storage. Given that FFPE blocks are typically transferred for testing immediately after diagnosis in clinical practice, DNA degradation is expected to be minimized. Because our classification algorithm begins with MMR IHC, cases with MMR-d and POLE-mut coexistence would be classified as MMR-d subgroup. These tumors are uncommon, accounting for only 5% of endometrial carcinomas, and data are limited [10]. Another potential limitation is the use of a limited MMR IHC panel (PMS2 and MSH6). Yet, when compared to the full panel, this method has a less than 0.5% chance of missing MMR-d [39] and this two-marker panel has now been proposed for clinical use in the 2023 FIGO staging system [10].

## Conclusions

Our study of molecular-based classification of endometrial carcinoma in Northern Thailand discovered that subgroup distribution and prognostic implications were comparable to the previous reports. These findings support the implementation of management guidelines that include molecular information in this region. At least *POLE* mutation testing in early-stage patients should be considered in resource-limited settings.

## Data Availability

The datasets used and/or analyzed during the current study are available upon reasonable request.

## References

[1] Bray F, Ferlay J, Soerjomataram I, Siegel RL, Torre LA, Jemal A (2018). Global cancer statistics 2018: GLOBOCAN estimates of incidence and mortality worldwide for 36 cancers in 185 countries. CA Cancer J Clin.

[2] Organization WH, WHO South-East Asia (SEARO) - Global Cancer Observatory: World Health Organization. ; 2020 [Available from: https://gco.iarc.fr/today/data/factsheets/populations/995-who-south-east-asia-searo-fact-sheets.pdf.

[3] Koskas M, Amant F, Mirza MR, Creutzberg CL (2021). Cancer of the corpus uteri: 2021 update. Int J Gynaecol Obstet.

[4] Gilks CB, Oliva E, Soslow RA (2013). Poor interobserver reproducibility in the diagnosis of high-grade endometrial carcinoma. Am J Surg Pathol.

[5] Han G, Sidhu D, Duggan MA, Arseneau J, Cesari M, Clement PB (2013). Reproducibility of histological cell type in high-grade endometrial carcinoma. Mod Pathol.

[6] Kandoth C, Schultz N, Cherniack AD, Akbani R, Liu Y, Cancer Genome Atlas Research N (2013). Integrated genomic characterization of endometrial carcinoma. Nature.

[7] Talhouk A, McAlpine JN (2016). New classification of endometrial cancers: the development and potential applications of genomic-based classification in research and clinical care. Gynecol Oncol Res Pract.

[8] Alexa M, Hasenburg A, Battista MJ. The TCGA Molecular classification of Endometrial Cancer and its possible impact on adjuvant treatment decisions. Cancers (Basel). 2021;13(6). 10.3390/cancers13061478 PMC8005218 33806979

[9] Concin N, Matias-Guiu X, Vergote I, Cibula D, Mirza MR, Marnitz S (2021). ESGO/ESTRO/ESP guidelines for the management of patients with endometrial carcinoma. Int J Gynecol Cancer.

[10] Berek JS, Matias-Guiu X, Creutzberg C, Fotopoulou C, Gaffney D, Kehoe S (2023). FIGO staging of endometrial cancer: 2023. Int J Gynaecol Obstet.

[11] Njoku K, Barr CE, Crosbie EJ (2022). Current and emerging prognostic biomarkers in Endometrial Cancer. Front Oncol.

[12] Tumours WCo. Female Genital Tumours / edited by WHO classification of Tumours Editorial Board. Fifth ed. Lyon: International Agency for Research on Cancer (IARC), 2020; 2020.

[13] Mojtahed A, Schrijver I, Ford JM, Longacre TA, Pai RK (2011). A two-antibody mismatch repair protein immunohistochemistry screening approach for colorectal carcinomas, skin sebaceous tumors, and gynecologic tract carcinomas. Mod Pathol.

[14] Stelloo E, Jansen AML, Osse EM, Nout RA, Creutzberg CL, Ruano D (2017). Practical guidance for mismatch repair-deficiency testing in endometrial cancer. Ann Oncol.

[15] Kobel M, Ronnett BM, Singh N, Soslow RA, Gilks CB, McCluggage WG (2019). Interpretation of P53 immunohistochemistry in Endometrial Carcinomas: toward increased reproducibility. Int J Gynecol Pathol.

[16] Billingsley CC, Cohn DE, Mutch DG, Stephens JA, Suarez AA, Goodfellow PJ (2015). Polymerase varepsilon (POLE) mutations in endometrial cancer: clinical outcomes and implications for Lynch syndrome testing. Cancer.

[17] Talhouk A, McConechy MK, Leung S, Yang W, Lum A, Senz J (2017). Confirmation of ProMisE: a simple, genomics-based clinical classifier for endometrial cancer. Cancer.

[18] Lambert PC (2009). Further development of flexible parametric models for survival analysis. Stata.

[19] Confounder summary score [Internet]. Wiley StatRef: Statistics Reference Online. 2015. Available from: https://onlinelibrary.wiley.com/doi/full/10.1002/9781118445112.stat05133.pub2.

[20] Royston PPMB (2011). The use of restricted mean survival time to estimate the treatment effect in randomized clinical trials when the proportional hazards assumption is in doubt. Stat Med.

[21] Talhouk A, McConechy MK, Leung S, Li-Chang HH, Kwon JS, Melnyk N (2015). A clinically applicable molecular-based classification for endometrial cancers. Br J Cancer.

[22] Stelloo E, Nout RA, Osse EM, Jurgenliemk-Schulz IJ, Jobsen JJ, Lutgens LC (2016). Improved Risk Assessment by integrating molecular and clinicopathological factors in early-stage endometrial Cancer-combined analysis of the PORTEC cohorts. Clin Cancer Res.

[23] Cosgrove CM, Tritchler DL, Cohn DE, Mutch DG, Rush CM, Lankes HA (2018). An NRG Oncology/GOG study of molecular classification for risk prediction in endometrioid endometrial cancer. Gynecol Oncol.

[24] Kommoss S, McConechy MK, Kommoss F, Leung S, Bunz A, Magrill J (2018). Final validation of the ProMisE molecular classifier for endometrial carcinoma in a large population-based case series. Ann Oncol.

[25] Henry CE, Phan K, Orsman EJ, Kenwright D, Thunders MC, Filoche SK. Molecular Profiling of Endometrial Cancer: an exploratory study in Aotearoa, New Zealand. Cancers (Basel). 2021;13(22). 10.3390/cancers13225641 PMC8615986 34830795

[26] Asami Y, Kobayashi Kato M, Hiranuma K, Matsuda M, Shimada Y, Ishikawa M (2023). Utility of molecular subtypes and genetic alterations for evaluating clinical outcomes in 1029 patients with endometrial cancer. Br J Cancer.

[27] Kim G, Lee SK, Suh DH, Kim K, No JH, Kim YB (2022). Clinical evaluation of a droplet digital PCR assay for detecting POLE mutations and molecular classification of endometrial cancer. J Gynecol Oncol.

[28] Jumaah AS, Al-Haddad HS, McAllister KA, Yasseen AA (2022). The clinicopathology and survival characteristics of patients with POLE proofreading mutations in endometrial carcinoma: a systematic review and meta-analysis. PLoS ONE.

[29] Van Gool IC, Ubachs JEH, Stelloo E, de Kroon CD, Goeman JJ, Smit V (2018). Blinded histopathological characterisation of POLE exonuclease domain-mutant endometrial cancers: sheep in wolf’s clothing. Histopathology.

[30] Leon-Castillo A, Gilvazquez E, Nout R, Smit VT, McAlpine JN, McConechy M (2020). Clinicopathological and molecular characterisation of ‘multiple-classifier’ endometrial carcinomas. J Pathol.

[31] Van den Heerik A, Ter Haar NT, Vermij L, Jobsen JJ, Brinkhuis M, Roothaan SM (2023). QPOLE: a quick, simple, and cheap alternative for POLE sequencing in Endometrial Cancer by Multiplex genotyping quantitative polymerase chain reaction. JCO Glob Oncol.

[32] Jamieson A, McConechy MK, Lum A, Leung S, Thompson EF, Senz J (2023). Harmonized molecular classification; assessment of a single-test ProMisE NGS tool. Gynecol Oncol.

[33] Li Y, Feng J, Zhao C, Meng L, Shi S, Liu K (2022). A new strategy in molecular typing: the accuracy of an NGS panel for the molecular classification of endometrial cancers. Ann Transl Med.

[34] Laczmanska I, Michalowska D, Jedryka M, Blomka D, Semeniuk M, Czykalko E (2023). Fast and reliable Sanger POLE sequencing protocol in FFPE tissues of endometrial cancer. Pathol Res Pract.

[35] Di Donato V, Giannini A, Bogani G. Recent advances in Endometrial Cancer Management. J Clin Med. 2023;12(6). 10.3390/jcm12062241 PMC10053513 36983243

[36] Golia D, Cuccu I, Santangelo G, Muzii L, Giannini A, Bogani G et al. Novel insights into Molecular Mechanisms of Endometrial Diseases. Biomolecules. 2023;13(3). 10.3390/biom13030499 PMC10046407 36979434

[37] Cuccu I, D’Oria O, Sgamba L, De Angelis E, Golia D’Auge T, Turetta C et al. Role of genomic and molecular Biology in the modulation of the treatment of Endometrial Cancer: Narrative Review and Perspectives. Healthc (Basel). 2023;11(4). 10.3390/healthcare11040571 PMC9957190 36833105

[38] Bogani G, Chiappa V, Lopez S, Salvatore C, Interlenghi M, D’Oria O et al. Radiomics and Molecular classification in Endometrial Cancer (the ROME Study): a Step Forward to a simplified Precision Medicine. Healthc (Basel). 2022;10(12). 10.3390/healthcare10122464 PMC9778151 36553988

[39] Aiyer KTS, Doeleman T, Ryan NA, Nielsen M, Crosbie EJ, Smit V (2022). Validity of a two-antibody testing algorithm for mismatch repair deficiency testing in cancer; a systematic literature review and meta-analysis. Mod Pathol.

